# Do No Harm: Potential Iatrogenic Harm of School Mental Health Interventions

**DOI:** 10.1192/j.eurpsy.2025.128

**Published:** 2025-08-26

**Authors:** J. Andrews

**Affiliations:** University of Oxford, Oxford, United Kingdom

## Abstract

**Abstract:**

In this talk I will discuss current findings related to therapeutically informed (e.g., Mindfulness and CBT based) universal school-based interventions for mental health. For example, I will highlight the evidence showing that many of these interventions often lead to null or unstained positive effects, have the potential to elicit negative effects and are not well liked by young people themselves. I will end by suggesting the field moves away from these universal interventions, towards more effective alternatives such as targeted, indirect and 1:1 interventions

**Disclosure of Interest:**

None Declared

